# Revisiting species delimitation within the genus *Oxystele* using DNA barcoding approach

**DOI:** 10.3897/zookeys.365.5356

**Published:** 2013-12-30

**Authors:** Herman Van Der Bank, Dai Herbert, Richard Greenfield, Kowiyou Yessoufou

**Affiliations:** 1 Department of Zoology, African Centre for DNA Barcoding (ACDB), Kingsway Campus, University of Johannesburg, PO Box 524, Auckland Park 2006, South Africa; 2 KwaZulu-Natal Museum, P. Bag 9070, Pietermaritzburg 3200, South Africa, and School of Life Sciences, University of KwaZulu-Natal, Pietermaritzburg, 3206 South Africa; 3 Department of Botany and Plant Biotechnology, African Centre for DNA Barcoding (ACDB), Kingsway Campus, University of Johannesburg, PO Box 524, Auckland Park 2006, South Africa

**Keywords:** Mollusca, Gastropoda, Trochidae, species delimitation, morphology

## Abstract

The genus *Oxystele*, a member of the highly diverse marine gastropod superfamily Trochoidea, is endemic to southern Africa. Members of the genus include some of the most abundant molluscs on southern African shores and are important components of littoral biodiversity in rocky intertidal habitats. Species delimitation within the genus is still controversial, especially regarding the complex *O. impervia* / *O. variegata*. Here, we assessed species boundaries within the genus using DNA barcoding and phylogenetic tree reconstruction. We analysed 56 specimens using the mitochondrial gene COI. Our analysis delimits five molecular operational taxonomic units (MOTUs), and distinguishes *O. impervia* from *O. variegata*. However, we reveal important discrepancies between MOTUs and morphology-based species identification and discuss alternative hypotheses that can account for this. Finally, we indicate the need for future study that includes additional genes, and the combination of both morphology and genetic techniques (e.g. AFLP or microsatellites) to get deeper insight into species delimitation within the genus.

## Introduction

Molluscs comprise one of the largest marine phyla, comprising more than 50 000 described species (marine species only), of which less than 10% are currently included in the global database of DNA barcodes (Radulovici et al. 2010). DNA barcoding is a genetic technique designed to standardize and accelerate species identification as an instrument facilitating conservation efforts, ecosystem monitoring, and the identification of phylogeographic and speciation patterns (Radulovici et al. 2010; but see Taylor and Harris 2012 for criticism). It has also proved valuable in population genetics and phylogenetic analyses, identification of prey in gut contents, forensic and seafood safety, invasion biology (Armstrong and Ball 2005, Bucklin et al. 2011) and in revealing cryptic species (Hebert et al. 2004, Puillandre et al. 2009, Lakra et al. 2011). One of the important uses of DNA barcoding is its ability to correctly assign several life-forms including larvae, carcass fragments and damaged specimens to species (Ward et al. 2005, Yang et al. 2012).

Although the mitochondrial cytochrome *c* oxidase I gene (COI), used for barcoding purposes of animals is not efficient for all taxonomic groups (e.g. terrestrial gastropods, Davison et al. 2009; anthozoans, Huang et al. 2008), and pending the integration of the next generation sequencing into the DNA barcoding technique (Taylor and Harris 2012), the barcoding approach has proved valuable in discriminating marine biodiversity (e.g. Sun et al. 2012; see also reviews in Radulovici et al. 2010). *Oxystele* Philippi, 1847, a genus of the highly diverse marine gastropod superfamily Trochoidea (Williams et al. 2010), is endemic to southern Africa. Currently, five species are recognised (Branch et al. 2010), but delimitation within the genus is still debated (Heller and Dempster 1991, Williams et al. 2010), especially due to strong homoplasy in morphological characters traditionally used in identification keys (Hickman 1998).

In this study, our main objective was to infer species boundaries within the genus using DNA barcode. To date, attempts to resolve taxonomic issues within the genus using DNA sequence data were very limited in sample size: only one individual of each of the five recognised *Oxystele* species was generally analysed. For this purpose, we sampled 56 specimens including all five *Oxystele* species from a wide geographic distribution range. We then applied the DNA barcoding approach for taxa delimitation.

## Materials and methods

### Sample collections

Sampling sites were widely distributed to cover the geographical distribution range of the genus. Species identification was done using the morphological characters given in the key to *Oxystele* species provided by Heller and Dempster (1991). Collection details including GPS coordinates, altitude and photographs of specimens are available online in the Barcode of Life Data Systems (BOLD; www.boldsystems.org) together with DNA sequences. Voucher specimens (shells) were also collected and deposited at the KwaZulu-Natal Museum (South Africa).

### DNA extraction, amplification and sequencing of DNA barcodes

DNA extraction, polymerase chain reactions (PCR) and sequencing of the COI region (animal DNA barcode) were done at the Canadian Centre for DNA Barcoding (CCDB). PCR reactions followed standard CCDB protocols as described by Hajibabaei et al. (2005). This results in 51 COI DNA sequences being generated. We also included in the DNA matrix five COI sequences that we retrieved from BOLD (DQ numbers in Table 1), making the total sequences analysed to a total of 56 COI sequences. Sequence alignment was performed using Multiple Sequence Comparison by Log-Expectation (MUSCLE vs. 3.8.31, Edgar 2004). GenBank accession numbers, BOLD process identification numbers and voucher information are all available online (www.boldsystems.org). These numbers, together with authorities for the species studied are listed in Table 1.

**Table 1.** Species, authority, GenBank accession numbers (*DQ*) and BOLD process ID numbers (HVDBM) of specimens studied. Specimens in bold are those for which morphological characters (weathered shell colours and patterns) failed to provide accurate identification; this is revealed in the barcoding test of species delimitation and in phylogenetic tree topology. Sample localities for *Oxystele impervia* and *Oxystele variegata* individuals are indicated: southern Cape^1^, Robben Island^2^, north-western Cape^3^, Namibia^4^

Species (authority):	GenBank and process ID numbers of specimens included in this study	Composition of MOTUs based on the barcoding test of species delimitation
*Oxystele sinensis* (Gmelin, 1791)	DQ061089, HVDBM056-10, HVDBM083-10, HVDBM084-10, HVDBM085-10, HVDBM086-10, HVDBM087-10, HVDBM409-11, HVDBM410-11, HVDBM411-11, HVDBM412-11, HVDBM437-11	DQ061089, HVDBM056-10, HVDBM083-10, HVDBM084-10, HVDBM085-10, HVDBM086-10, HVDBM087-10, HVDBM409-11, HVDBM410-11, HVDBM411-11, HVDBM412-11, HVDBM437-11
*Oxystele tabularis* (Krauss, 1848)	DQ061090, HVDBM289-11, HVDBM338-11, HVDBM339-11	DQ061090, HVDBM289-11, HVDBM338-11, HVDBM339-11
*Oxystele tigrina* (Anton, 1838)	DQ061091, HVDBM005-10, HVDBM006-10, HVDBM013-10, HVDBM055-10, HVDBM394-11, HVDBM506-11, HVDBM507-11, HVDBM508-11, HVDBM509-11, HVDBM510-11	DQ061091, HVDBM005-10, HVDBM006-10, HVDBM013-10, HVDBM055-10, HVDBM394-11, HVDBM506-11, HVDBM507-11, HVDBM508-11, HVDBM509-11, HVDBM510-11
*Oxystele variegata* (Anton, 1838)	DQ061092^1^, HVDBM058-10^1^, HVDBM059-10^1^, HVDBM070-10^1^, HVDBM072-10^1^, HVDBM183-10^3^, HVDBM184-10^3^, HVDBM185-10^3^, HVDBM208-10^4^, HVDBM209-10^4^, HVDBM389-11^3^, HVDBM393-11^1^, HVDBM395-11^1^, HVDBM456-11^4^, HVDBM457-11^4^, HVDBM511-11^2^, HVDBM512-11^2^, HVDBM513-11^2^, HVDBM514-11^2^, HVDBM515-11^2^	HVDBM072-10^1^, HVDBM183-10^3^, HVDBM184-10^3^, HVDBM185-10^3^, HVDBM208-10^4^, HVDBM209-10^4^, HVDBM389-11^3^, HVDBM393-11^1^, HVDBM395-11^1^, HVDBM456-11^4^, HVDBM457-11^4^, HVDBM511-11^2^, HVDBM512-11^2^, HVDBM513-11^2^, HVDBM514-11^2^, HVDBM515-11^2^, **HVDBM028-10**^1^
*Oxystele impervia* (Menke, 1843)	DQ061093^1^, HVDBM022-10^1^, HVDBM027-10^1^, HVDBM028-10^1^, HVDBM057-10^1^, HVDBM071-10^1^, HVDBM178-10^3^, HVDBM179-10^3^, HVDBM180-10^3^	**DQ061093**^1^, HVDBM022-10^1^, HVDBM027-10^1^, HVDBM057-10^1^, HVDBM071-10^1^, HVDBM178-10^3^, HVDBM179-10^3^, HVDBM180-10^3^<br/> **DQ061092**^1^, **HVDBM058-10**^1^, **HVDBM059-10**^1^, **HVDBM070-10**^1^

### Data analysis

We assessed the “DNA barcode gap” (Meyer and Paulay 2005) in the dataset using two approaches. First, we compared the median of interspecific distances with that of intraspecific distances (genetic distances are calculated between morphospecies). Significance of the differences between both distances was assessed using the non-parametric Wilcoxon ranked sum test. Second, we used Meier et al.’s (2008) approach, that is, we compared the smallest interspecific distance with the largest intraspecific distance. Genetic distances were measured using the Kimura 2-parameter (K2P) model (Kimura 1980). We are aware of the recent literature indicating that the K2P-model might not be the best model for DNA barcoding. However, we used this model here to allow comparison of our results with other DNA barcoding studies where K2P-model is the most frequently used model.

We also tested the discriminatory power of DNA barcoding by evaluating the proportion of correct species identification using the COI region. All sequences were labeled according to the names of the species from which the sequences were generated. The test of discriminatory power works as follows. Each sequence is considered as an unknown while the remaining sequences in the dataset are considered as the DNA barcode database used for identification. If the identification of the query is the same as the pre-considered identification (i.e. the sequence labels), the identification test is scored as “correct”, and the overall proportion of correct identification corresponds to the discriminatory power of the region tested, i.e. COI. This test was done applying three approaches: the “best close match” (Meier et al. 2006), the “near neighbour” and the BOLD criteria using respectively the functions bestCloseMatch, threshID, and nearNeighbour implemented in the program Spider v1.1-1 (Brown et al. 2012). Prior to the test, we determined the optimised genetic distance suitable as threshold for taxon identification. For this purpose, we used the function localMinima also implemented in Spider (Brown et al. 2012).

The function bestCloseMatch conducts the “best close match” analysis of Meier et al. (2006), searching for the closest individual in the dataset. If the closest individual is within a given threshold, the outcome is scored as “correct”. If it is further than the given threshold, the result is “no ID” (no identification). If more than one species are tied for closest match, the outcome of the test is “ambiguous” identification. When all matches within the threshold are different species to the query, the result is scored as “incorrect”.

The function threshID conducts a threshold-based analysis based on a threshold genetic distance of 1% as conducted by the “Identify Specimen” tool provided by the BOLD system (http://www.boldsystems.org/views/idrequest.php). It is more inclusive than bestCloseMatch, in that it considers all sequences within the threshold of 1%. There also four possible outcomes for threshID tests, that is, “correct”, “incorrect”, “ambiguous”, and “no id” similar to the outcomes of the bestCloseMatch function.

The nearNeighbour function finds the closest individual and returns the score “true” (equivalent to “correct”) if their names are the same, but if the names are different, the outcome is scored as “false” (equivalent to “incorrect”).

Further, we performed a barcoding test of taxon delimitation. In reality, this test groups specimens into “molecular operational taxonomic units” (MOTUs; Jones et al. 2011), which are generally regarded as proxy for morpho-species (Stahlhut et al. 2013). MOTUs are defined as groups of specimens that are within the genetic threshold used for taxon delimitation. If all specimens of the same morpho-species are clustered in a single MOTU, this means that MOTUs are congruent with morpho-species, thus increasing the taxonomic value of DNA barcoding. The delimitation of MOTUs was conducted using the function tclust in the R package Spider v1.1-1. If two specimens are more distant than the threshold from each other, but both are within the threshold of a third, the function tclustidentifiedall three individuals as a single MOTU. We also identified the composition of each MOTU using the function lapply also implemented in Spider.

Finally we complemented the test of MOTU delimitation with a phylogenetic analysis of COI sequences. We reconstructed a phylogenetic tree using Bayesian and maximum parsimony methods. The Bayesian tree was reconstructed using MrBayes v3.1.2 (Ronquist and Huelsenbeck 2003). The best-fit model of DNA sequence evolution was chosen using jModelTest v0.1.1 (Posada 2008) under the Akaike information criterion (Posada and Buckley 2004). The TrN + I model was selected and used to generate the Bayesian tree. Analysis was run for nine million generations with sampling one tree every 100 generations. Two independent Bayesian analyses with four differentially heated chains were performed simultaneously. The results were visualised and checked using MEGA, and 25 000 trees were discarded as burn-in to ensure that the analysis had stabilised. Node support was assessed using posterior probability (PP) as follows: PP > 0.95: high support and PP < 0.95: no support (Alfaro and Holder 2006).

Maximum parsimony (MP) was implemented to analyse the data using PAUP* v4.0b10 (Swofford 2002). Tree searches were done using heuristic searches with 1 000 random sequence additions but keeping only 10 trees. Tree bisection-reconnection was performed with all character transformations treated as equally likely i.e. Fitch parsimony (Fitch 1971). MP searches and bootstrap resampling (Felsenstein 1985) were done using PAUP* v4.0b10 (Swofford 2002).

*Jujubinus exasperatus* (Pennant, 1777) was used as outgroup based on Williams et al. (2010). Node support was assessed using bootstrap (BP) values: BP > 70% for strong support (Murphy et al. 2001, Wilcox et al. 2002).

## Results

Our dataset includes 56 specimens: nine specimens of *Oxystele impervia*, 12 of *Oxystele sinensis*, four of *Oxystele tabularis*, 11 of *Oxystele tigrina*, and 20 specimens of *Oxystele variegata* (Table 1). The aligned COI matrix was 654 base pairs in length, including A: 24.2%; C: 21.1%; G: 18.3% and T: 36.4%.

**Figure 1.**
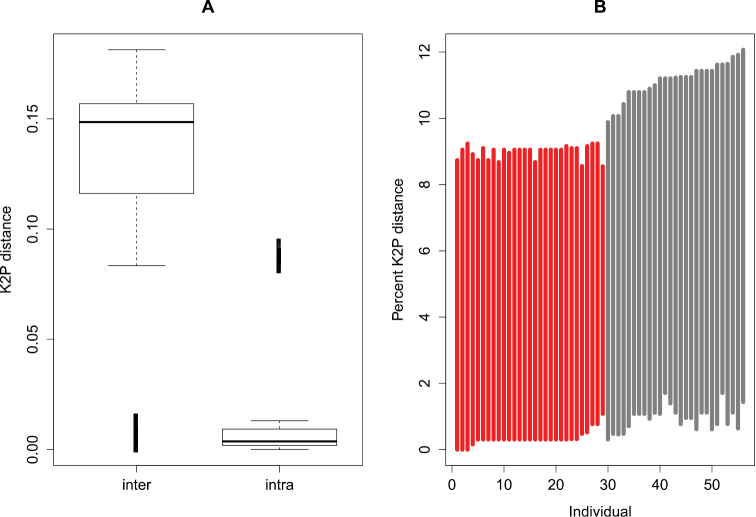
Evaluation of barcode gap in the dataset. **A** Boxplot of the interspecific (inter) and intraspecific genetic (intra) distances, indicating the existence of a barcode gap i.e. intraspecific distance is longer than intraspecific distance. The bottom and top of the boxes show the first and third quartiles respectively, the median is indicated by the horizontal line, the range of the data by the vertical dashed line and outliers (points outside 1.5 times the interquartile range) by Bold vertical lines **B** Lineplot of the barcode gap for the 56 *Oxsystele* specimens. For each specimen in the dataset, the grey lines indicate where the smallest interspecific distance (top of line value) is longer than the longest intraspecific distance (bottom of line value), therefore indicating existence of barcode gap; the red lines show where this pattern is reversed, and the closest non-conspecific is closer to the query than its nearest conspecific, i.e., the situation where there is no barcoding gap.

Interspecific distances range from 0 to 0.18 (median = 0.15) and are generally larger than intraspecific distances (range: 0-0.09; median = 0.004; Wilcoxon test, p < 0.001; Figure 1A). This indicates that there is a barcode gap in the dataset. Even when we compared the lowest interspecific versus the furthest intraspecific distance, we also found that barcode gap exists within the COI sequences (grey lines in Figure 1B).

**Figure 2.**
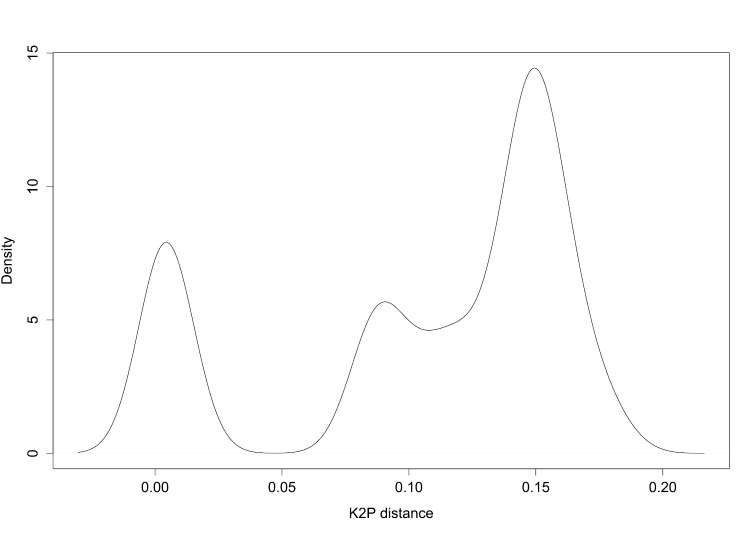
Determination of the threshold genetic distance for species identification. The density plot indicates transition between intra- and interspecific distances; the genetic distance corresponding to this transition (dip in the density graph, here approximately 0.05) indicates the suitable threshold to the dataset. This method does not require prior knowledge of species identity to get an indication of potential threshold values.

We determined the optimised threshold genetic distance (d) with which we tested the discriminatory power of COI sequences and delimited MOTUs. We found d = 0.047 (Figure 2). Testing the efficacy of DNA barcoding based on this threshold, we found that COI sequences performed very well in assigning DNA sequences to the correct species (Table 2). For instance, under both near neighbour and best close match methods, 87.5% of the COI sequences were correctly identified (49 specimens out of 56). However, the best close match method indicates 5.36% of ambiguity (three specimens), i.e. both correct and incorrect species are within the given threshold; and 7.14% of incorrect identification (four specimens). Also, for 12.5% of sequences (seven specimens) the near neighbour method results in “incorrect”. Using the BOLD method (threshold = 1%), we obtained poor barcoding performance, that is, we have as many correct as ambiguous results (48.21% respectively; i.e. 27 specimens). The BOLD method also indicates one “incorrect” and one “no id” (Table 2).

**Table 2.** Tests of barcoding identification accuracy with numbers (n) and percentages (%) of each score.

Methods	Near neighbour		Best Close match				BOLD criteria			
Scores	False	True	Ambiguous	Correct	Incorrect	No ID	Ambiguous	Correct	Incorrect	No ID
n (%)	7 (12.5%)	49 (87.5%)	3 (5.36%)	49 (87.5%)	4 (7.14%)	0 (0%)	27 (48.21%)	27 (48.21%)	1 (1.79%)	1 (1.79%)

**Figure 3.**
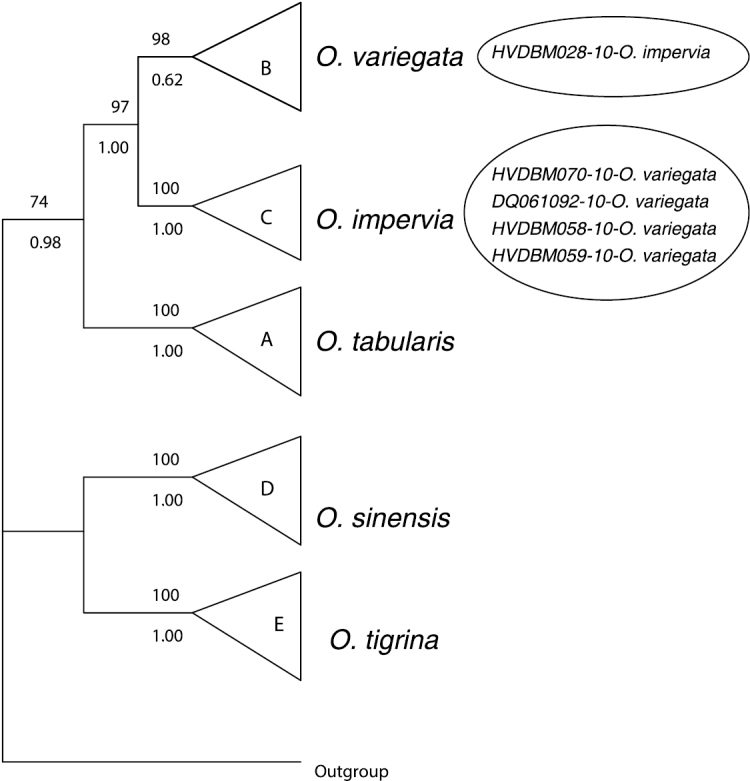
Summary of both Bayesian and parsimonious trees. Values above branches indicate bootstrap supports; values under branches indicate posterior probability. All distinguished species are indicated at the tip of the tree. Branches without values indicate non-supported nodes; the small circle indicates a specimen of *Oxystele impervia* (HVDBM028-10) that was misidentified based on morphology; large circle indicates four specimens morphologically indistinguishable from *Oxystele variegata* (HVDBM070-10; DQ061092; HVDBM058-10; HVDBM059-10), but that are, based on both barcoding analysis of species delimitation (see Table 1) and phylogenetic tree analysis identified as *Oxystele impervia* (see also Appendices 1 and 2).

Further, all the 56 specimens included in this study were grouped into five MOTUs based on our threshold (Table 1). Using tree-based analysis, we also found five strongly supported groupings (PP = 1.00; BP = 100%), identified as A–E (Figure 3), except that the grouping B corresponding to *Oxystele variegata* is only well supported in the MP analysis (BP = 98%). The composition of these five groupings matches that of MOTUs and comprises *Oxystele tabularis* (A), *Oxystele variegata* (B), *Oxystele impervia* (C), *Oxystele sinensis* (D), and *Oxystele tigrina* (E) (Figures 3, Appendices 1 and 2).

## Discussion

The concept of DNA barcoding was first proposed as a technique to accelerate species identification within micro-organisms (Nanney 1982). However, it has now been generalised as a potential method that can help characterise and discover new species in broader taxonomic groups (Hebert et al. 2004, Van der Bank et al. 2012). In the animal kingdom, the COI region has proved valuable as a DNA barcode for many taxonomic groups, but it can also be problematic for others (Moritz 2004, Ebach and Holdrege 2005, Schindel and Miller 2005, Köhler 2007, Huang et al. 2008).

We first tested COI’s potential as a good barcode for the genus *Oxystele*. A good barcode candidate is expected to exhibit a barcode gap (Meyer and Paulay 2005), i.e. higher genetic variation between than within species (Hebert et al. 2003). Various options are currently available to evaluate the barcode gap. We used two approaches. We compared the median of interspecific versus intraspecific distances. We found that interspecific distance is significantly greater than intraspecific distance, suggesting that there is a barcode gap in COI data. We also applied the approach of Meier et al. (2008); i.e. compared the smallest interspecific versus the greatest intraspecific distances), rather than comparing just the median distances. This approach also reveals existence of a barcode gap, thus confirming COI as a potential DNA region for taxon identification within *Oxystele*. This DNA region has also proved successful for barcoding identification in other mollusc taxonomic groups (Davison et al. 2009, Köhler and Glaubrecht 2009, Feng et al. 2011a, b, Sun et al. 2012; but see Sauer and Hausdorf 2012 for limitation of single-locus DNA sequences).

In addition, we found that COI has a strong discriminatory power (85%) within the genus *Oxystele* especially using the best close match and near neighbour methods. This gives support to the efficacy of COI for identification purposes within the genus. However, the application of BOLD identification criteria yields a poor identification success i.e. < 50% and similar proportion of ambiguity (Table 2). The poor performance of COI using BOLD criteria should not be seen as a result of barcoding inefficiency, but should rather be linked to the untested 1% threshold used in BOLD identification (see Meyer and Paulay 2005).

Our analysis of barcoding-based taxon delimitation results in five MOTUs, of which three correspond to morphology-delimited species: *Oxystele sinensis*, *Oxystele tabularis* and *Oxystele tigrina* (Table 1).These results are also supported by phylogeny-based analysis of species delimitation. However, four specimens identified morphologically as *Oxystele variegata* are included by the barcoding taxon delimitation test within the MOTU of *Oxystele impervia*. Similarly, one specimen identified morphologically as *Oxystele impervia* is grouped within the MOTU of *Oxystele variegata* (Figure 3). These mismatches between morpho-species identification and barcoding-based taxon delimitation (MOTUs) reflect the controversy surrounding species boundaries and/or the identification key (e.g. Heller and Dempster’s (1991) key) currently used to distinguish the *impervia/variegata* complex.

Why the mismatch between MOTU and morpho-species? Potential explanations include unsuitable morphology-based taxon delimitation, species paraphyly (– including but not restricted to ancestral polymorphism), and on-going gene flow (i.e., the two taxa are not distinct species or they hybridize; see Funk and Omland 2003). Specifically, Funk and Omland (2003) demonstrated that about 25% of animal species are para- or even polyphyletic, suggesting that the non-monophyly of *Oxystele variegata* and *Oxystele impervia* in the examined gene tree is not necessarily an argument against their species status. This provides further evidence of the limitations of DNA barcoding in general. It is also possible that the rate of speciation events is slower or greater than that of morphological differentiation; e.g. rapid morphological changes can occur with little or no evolutionary changes (Adams et al. 2002); and this could be driven for example by habitat specialisation (Collar et al. 2010).

In our attempt to resolve the taxonomic uncertainty, we also used the phylogenetic tree reconstruction. The results are similar to those of MOTUs, that is, one specimen morphologically identified as *Oxystele impervia*, grouped on the phylogeny with *Oxystele variegata* (grouping B, Figure 3, Appendices 1 and 2), but this grouping B has strong support only in MP analysis.

The controversy regarding the complex has been reported in previous studies (Heller and Dempster 1991, Williams et al. 2010), likely reflecting the limitations in morphological characters (Hickman 1998) on which the current identification key is based. Heller and Dempster (1991) reported that *Oxystele impervia* and *Oxystele variegata* should be considered as two different species based on shell colour, radula cusp indentation, ecological (*Oxystele impervia* occurs higher up the shore than *Oxystele variegata*), and fixed allozyme differences at one enzyme-coding locus (out of 22). However, the overlaps in ecological zones and interspecific overlap of up to 66% in radula cusp indentation (Heller and Dempster 1991) indicate that these criteria (ecology and radula indentation) might be unreliable for taxon identification.

**Figure 4.**
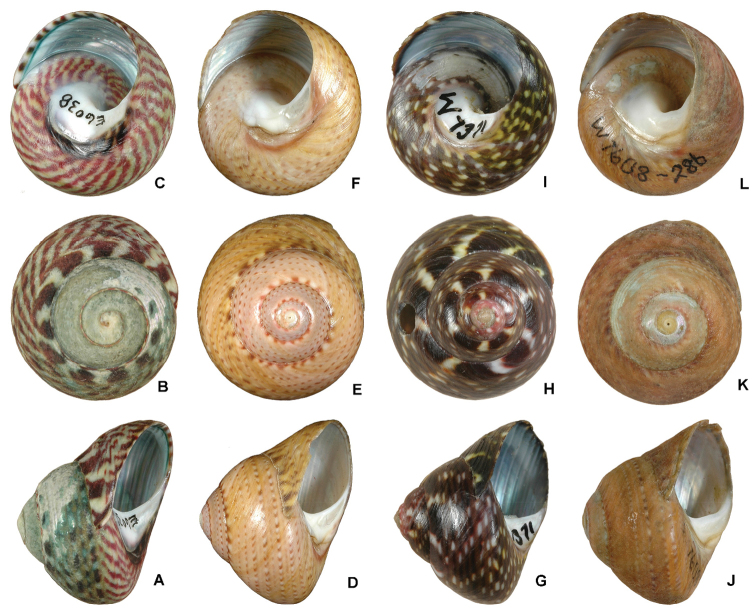
Patterns of shell colour within the genus *Oxystele*. **A–C** *Oxystele variegata* from Namibia, 5 km north of Swakopmund, diameter 22.2 mm (NMSA E6038) **D–F** *Oxystele impervia* from the Western Cape, Groen Rivier, diameter 22.3 mm (NMSA E7353) **G–I** *Oxystele* sp. from theEastern Cape, Tsitsikamma National Park, diameter 16.5 mm (HVDBM058-10, NMSA W7371); the colour pattern of these specimens suggests *Oxystele variegata*, but these specimens group within the unit of *Oxystele impervia* **J–L** *Oxystele* sp. from the Northern Cape, Noup, diameter 18.0 mm (HVDBM185-10, NMSA W7608); the colour pattern suggests *Oxystele impervia*, but they group with *Oxystele variegata* (see Figure 4 and Appendix 2 for the phylogenetic groupings of these specimens and node supports; these groupings contradict their morphological identification).

In addition, Heller and Dempster (1991) described 24 different photos of shell colours and patterns of typical *Oxystele impervia* and *Oxystele variegata* (12 photos for each species), but the differentiation they proposed is still unclear and could lead to multiple interpretations as indicated in the words such as “very infrequently”, “off-white”, or “greenish-grey” and “almost never” that they used to distinguish between both species. Also, overlaps in colours and weathered shells make Heller and Dempster’s (1991) keys unreliable to identify some individuals (e.g. see Figure 4). Specimens of both *Oxystele impervia* and *Oxystele variegata* are commonly weathered to some extent, resulting in shell colour being indistinct or scarcely discernible. Some specimens (e.g. as shown in Figure 4) can only be tentatively identified because they exhibit unusual colour patterns, not clearly consistent with published photos in Heller and Dempster (1991).

Williams et al. (2010) however suggested that *Oxystele impervia* and *Oxystele variegata* should be regarded as one species based on analysis from a single individual from each species. DH inspected the morphology of the samples (available on MorphoBank) used in the study by Williams et al. (2010) and confirmed that the shell of specimen DQ061092 is very typical of that of *Oxystele variegata*, but that DQ061093 has a more intermediate form with a finer colour pattern. He concluded that the latter is not obviously referable to any one of *Oxystele impervia* and/or *Oxystele variegata*, more than to the other. In this study, the fact that both specimens come out not only on the phylogeny in the grouping of *Oxystele impervia* (grouping C on the phylogeny; with strong support from PP and BP; Figures 3, Appendices 1 and 2), but also in the MOTU delimitation (Table 1), is surprising (particularly DQ061092, which is morphologically typical of *Oxystele variegata*).

One of six polymorphic loci (glycyl-leucine peptidase or peptidase A; Van der Bank 2002) indicated fixed allele differences between *Oxystele impervia* and *Oxystele variegata*, and this was the most convincing characteristic to differentiate between both species (Heller and Dempster 1991). Williams et al. (2010) argue that differences in allele frequency could result from selection pressures (e.g. peptidase in *Mytilus*; Hilbish 1985). They further indicate that differences in habitat preferences, as reported for the *impervia*/*variegata* complex, could subject them to variation in salinity or temperature, which could lead to variation not only in diets but also in allozymes and morphology.

Indeed morphological differentiation between both species can be difficult. Some of the shell colours and patterns are similar, and radula morphology could be altered as a result of differences in diet, age and other factors. For example, Padilla (1998) demonstrated that two species of Gastropoda “produce differently shaped teeth when fed different foods, displaying intraspecific variability as extreme as would usually be considered to define different species”. Such variation in morphological characters has also been reported to be misleading in other groups such as spiders where the description of almost 50% of the known species was mistakenly based on the same species (Coddington and Levi 1991). Indeed molluscs are well-known to exhibit considerable intraspecific variation in shell morphology (Colgan et al. 2007; Figure 4), and high adaptive capacity to various environmental conditions, leading to striking ecological, morphological and behavioural disparity among specimens within the same species (Ponder et al. 2008).

In this study, most of the specimens that group within unexpected MOTUs were collected from different localities, suggesting possible shell colour variation due to variation in environmental conditions. For example, specimens of *Oxystele variegata* from Namibia and Robben Island clustered on the phylogeny, but those from north-western and southern Africa (Cape) did not. The Cape is renowned for its bad weather as indicated in its common name of “The Cape of Storms”, resulting in weathering of individuals (i.e. see “Ships in trouble in Cape waters”; http://www.e-gnu.com/shipwreck_update.html).

## Conclusion

The split we found on the phylogeny and species delimitation analyses between *Oxystele impervia* and *Oxystele variegata* does not correspond with the nominal, morphologically-based identifications, indicating the need for the combination of morphological features and genetic data for further analysis. It is also possible that the COI gene alone is insufficient to discriminate species within the genus. We therefore suggest that future analysis should use a multi-gene approach. However, Donald et al. (2005) have studied three genes including two mitochondrial (16S + COI) and one nuclear (actin), and Williams et al. (2010) used one nuclear and three mitochondrial genes; but neither study was successful in teasing apart both species. We would therefore suggest that additional techniques such as AFLP or microsatellites should be applied in an attempt to reveal the status of *Oxystele impervia* and *Oxystele variegata*. Nevertheless, our analyses using barcoding confirm the existence of five MOTUs (probably suggestive of five species), with *Oxystele variegata* being a distinct species from *Oxystele impervia*.

## Figures and Tables

**Figure S1.**
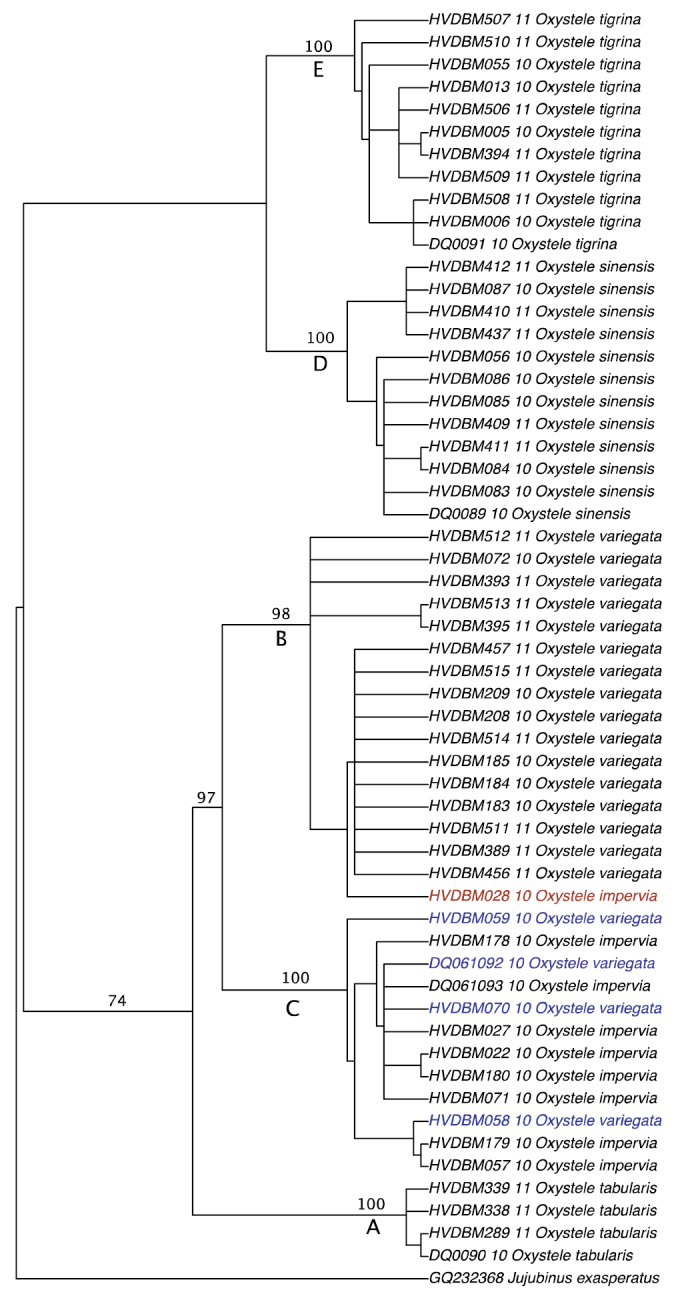
The only parsimonious tree obtained from the maximum parsimony (MP) analysis. Topology of species groupings is similar to that of the Bayesian tree (see Figure 3). Node supports are reported on the branches; the first value is bootstrap support from MP analysis; the second value in bracket indicates the posterior probability obtained from Bayesian analysis; only moderate to high node support values are indicated; *Jujubinus exasperatus* is used as outgroup; A-E indicates different possible species-units in the dataset: A (*Oxystele tabularis*), B (*Oxystele variegata*), C (*Oxystele impervia*), D (*Oxystele sinensis*), E (*Oxystele tigrina*), as in Figure 3.

**Figure S2.**
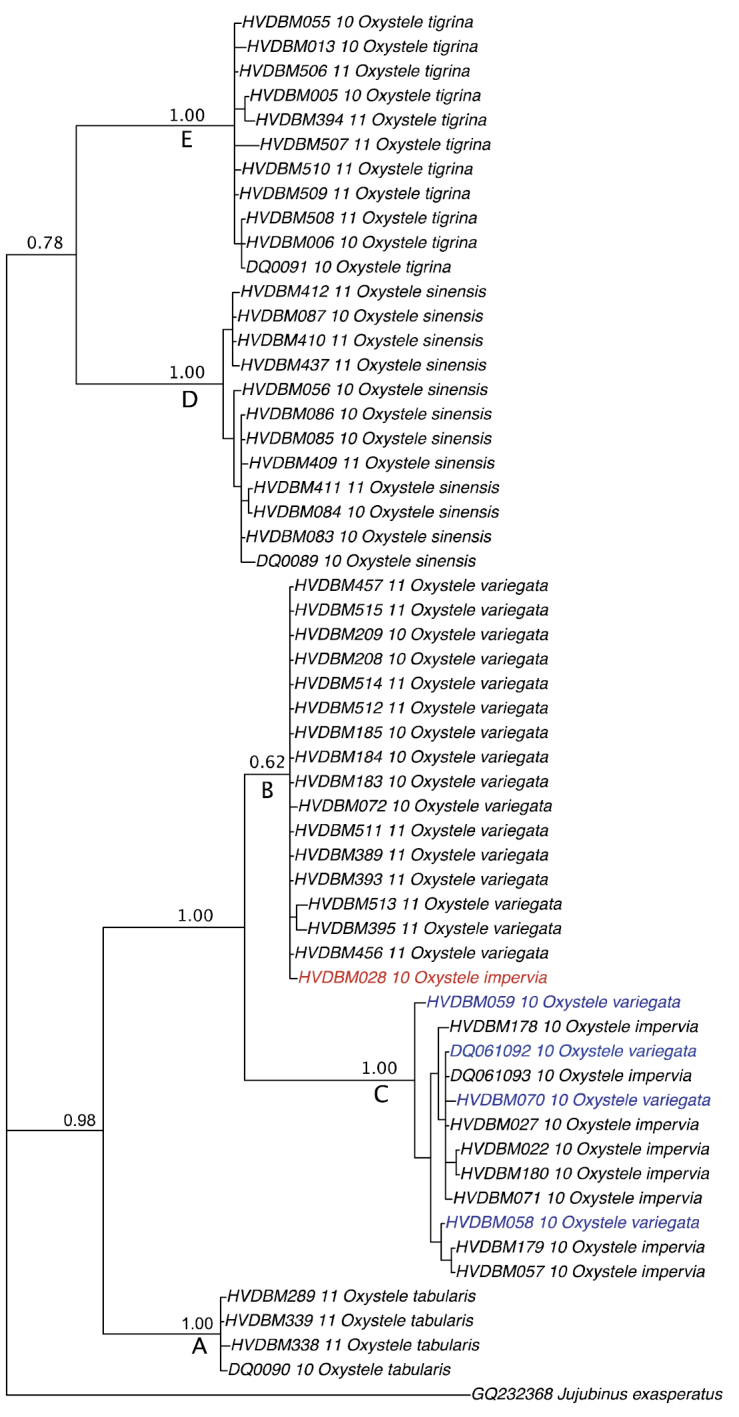
Bayesian tree assembled using MrBayes indicating the groupings of specimens and the posterior probability of the nodes.
